# Genome-Wide Identification and Expression Analysis of the Mediator Complex Subunit Gene Family in Cassava

**DOI:** 10.3390/ijms26041666

**Published:** 2025-02-15

**Authors:** Lingling Zhou, Shuhui Sun, Linlong Zhu, Xian Chen, Ran Xu, Lian Wu, Shuang Gu

**Affiliations:** 1 School of Breeding and Multiplication, Sanya Institute of Breeding and Multiplication, Hainan University, Sanya 572025, China; m15237352151@163.com (L.Z.); yx13515281250@163.com (S.S.); zhull22@126.com (L.Z.); datoua1116@163.com (X.C.); xuran@hainanu.edu.cn (R.X.); 2 School of Tropical Agriculture and Forestry, Hainan University, Haikou 570228, China

**Keywords:** Mediator complex subunit, cassava, abiotic stress, expression profile, gene family

## Abstract

The Mediator complex (MED) functions as a co-activator in plants, transmitting transcriptional signals to regulate gene expression, including responses to environmental stresses. While the MED gene family has been identified in several species, it has not yet been reported in cassava. In this study, we identified 32 members of the *MeMED* gene family in cassava (*Manihot esculenta* Crantz) distributed across 13 chromosomes. These genes were categorized into distinct Mediator subunits based on their similarity to *Arabidopsis* modules. Promoter analysis revealed the presence of various cis-regulatory elements, which likely play key roles in regulating plant growth, development, and stress responses. RNA-seq data showed tissue-specific expression patterns for the *MeMED* genes, with significant expression observed in leaves, roots, petioles, stems, friable embryogenic callus, and shoot apical meristems. Further RT-qPCR analysis under various abiotic stress conditions—including drought, exogenous hydrogen peroxide, cold, heat, and salt—demonstrated that 10 selected *MeMED* genes exhibited significant differential expression, indicating their potential functional involvement in stress adaptation. These findings offer insights into the biological roles of the *MeMED* gene family in cassava, with implications for improving stress tolerance in future breeding programs.

## 1. Introduction

The Mediator complex is an evolutionarily conserved multi-protein complex found across eukaryotes, playing a crucial role in regulating plant growth, development, defense, and hormone signaling [1,2]. It facilitates transcription by recruiting RNA polymerase II (Pol II) to specific gene promoters, linking transcription factors (TFs) bound at activators and repressors to the pre-initiation complex (PIC). Upon receiving regulatory signals, the Mediator complex undergoes conformational changes, creating a flexible surface that helps assemble the PIC. Acting as a molecular bridge, Mediator interacts with both the PIC and TFs to promote transcriptional activation [3,4].

Structurally, the core Mediator complex is divided into three main modules, namely the head, middle, and tail modules [5]. Each module comprises distinct subunits, contributing to the unique functional roles in transcription regulation [6]. While the number of subunits varies across species, plants typically have around 34 Mediator subunits [7]. The head module mainly interacts with Pol II to facilitate transcription, while the tail module is integral for binding gene-specific TFs. The middle module is responsible for transferring transcriptional signals between the tail and head modules and may also interact with Pol II directly [8]. Additionally, the kinase module, known as the CDK8 module, consists of CDK8, C-type cyclin (CycC), MED12, and MED13.

In recent years, multiple studies have highlighted the essential role of the Mediator complex in activating gene transcription in plants, particularly under environmental stresses such as salt, drought, cold, and heat [9,10,11]. Among its subunits, MED25 plays a positive role in salt stress responses by activating salt stress-responsive genes through interactions with key regulatory proteins, including DREB2A, ZINC FINGER HOMEODOMAIN 1 (ZFHD1), and MYB-like. The function of *MED25* in salt tolerance appears to be conserved across land plants. For instance, knocking out *PpMED25A* in Physcomitrella patens (a moss) increases the plant’s sensitivity to salt stress [12]. Similarly, the *med18* mutant in Arabidopsis exhibits salt sensitivity, and MED18 interacts with NUCLEOPORIN85 (NUP85) to regulate stress-responsive genes [13]. A recent study identified four Mediator subunits—MED9, MED16, MED18, and CDK8—each representing a distinct module, as essential regulators of transcriptional responses to salt and heat stress in Arabidopsis, as revealed by RNA-seq analysis [14]. The *med19a* mutant exhibits reduced drought resistance, characterized by faster water loss from its leaves and a lower survival rate under drought conditions [15]. Additionally, *MED16*, *MED14*, and *MED2* are crucial for activating the ABRE motif in response to ABA signaling. Mutants of these subunits are insensitive to ABA-induced growth inhibition and lose water more rapidly than wild-type plants, highlighting their specific role in drought adaptation [16]. Recent studies have also linked *CDK8* to drought stress regulation. Mutations in *CDK8* lead to increased stomatal density, impaired stomatal aperture control, and reduced drought tolerance. Conversely, the overexpression of *CDK8* enhances drought resistance [17].

Among the Mediator subunits, *MED16* is one of the earliest identified components involved in abiotic stress responses. It plays a critical role in cold stress tolerance by initiating physiological and metabolic adjustments that sustain cellular homeostasis. Before being classified as part of the Mediator complex, *MED16* was referred to as *SENSITIVE TO FREEZING6* (*SFR6*) and was recognized for its contribution to cold acclimation and enhanced freezing tolerance [18]. Additionally, *VuMED16* genes in asparagus bean have been found to influence cell wall modifications under cold stress, suggesting that the function of *MED16* in cold stress responses may be evolutionarily conserved across different plant species [19]. Further investigations into the rao1 mutant of Arabidopsis, which carries a mutation in *CDK8*, have revealed that *CDK8* is also involved in mitochondrial retrograde signaling under H_2_O_2_-induced oxidative stress and cold stress conditions [20].

More recently, the Mediator complex has been implicated in heat stress responses. In Arabidopsis, *MED14* and *MED17* mutants exhibit lower survival rates after heat treatment, reduced heat tolerance, and impaired acquired thermotolerance. Notably, approximately 25% of heat stress-induced genes are significantly downregulated in these mutants, further emphasizing the role of the Mediator complex in heat stress adaptation [21].

Overall, these studies collectively highlight the crucial role of the Mediator complex and its associated subunits in plant responses to abiotic stresses, including salt, drought, cold, and heat. Further research is needed to elucidate the precise molecular mechanisms through which these Mediator subunits coordinate stress adaptation in plants.

Cassava (*Manihot esculenta* Crantz), a member of the *Euphorbiaceae* family, is the sixth-largest food crop globally [22] and the third-largest source of caloric energy [23]. It provides essential dietary energy to nearly one billion people in tropical regions, including Africa and Latin America, playing a vital role in smallholder economies and food security. Known for its tolerance to drought and poor soils, cassava has become increasingly important as a climate-resilient crop in response to growing population pressures. However, improving cassava yield and quality remains challenging due to both biotic and abiotic stresses, as well as limited breeding technologies.

Members of the *MED* gene family have been relatively well characterized in species such as *Arabidopsis*, rice, and asparagus bean through genomic analysis [7,19]. However, the *MeMED* gene family in cassava has not been thoroughly studied. In this study, we conducted a comprehensive analysis of the *MeMED* gene family in cassava using assembled genome data. We examined the phylogenetic relationships, gene structures, chromosomal locations, synteny, and cis-elements to better understand their evolutionary patterns and potential functions. Additionally, we explored the expression profiles of *MeMED* genes across various cassava tissues using publicly available transcriptome data. We also assessed the responses of *MeMED* genes to different abiotic stresses through quantitative real-time RT-PCR (qRT-PCR). Our findings provide valuable insights that will guide the future functional characterization of *MeMED* genes in cassava.

## 2. Results

### 2.1. Identification of the MeMED Gene Family in Cassava

Through BlastP sequence alignment and querying the Conserved Domains Database (CDD), we identified a total of 32 members of the *MeMED* gene family in the cassava genome (Table 1). These 32 *MeMED* genes are distributed across 13 chromosomes (LG1, LG3, LG4, LG5, LG6, LG8, LG9, LG11, LG13, LG14, LG15, LG16, and LG17), and were named *MeMED33_3* to *MeCdk8_1* based on their *Arabidopsis* homologs (Table 1). The physicochemical characteristics of the 32 *MeMED* genes are summarized in Table 1.

The CDS lengths of these genes ranged from 318 bp (*MeMED28_2*) to 6798 bp (*MeMED12_1*), with an average CDS length of 2004 bp. Corresponding amino acid lengths varied from 106 (MeMED28_2) to 2266 (MeMED12_1), with an average length of 668.22 amino acids. The molecular weights of the proteins ranged from 12.41 kD (MeMED28_2) to 250.6 kD (MeMED12_1), with an average weight of 73.33 kD. The theoretical isoelectric points (pI) ranged from 4.4 (MeMED21_1) to 9.28 (MeMED31_1), with 24 proteins (75%) having a pI lower than seven, indicating they are weakly acidic, and eight proteins (25%) having a pI greater than seven, indicating they are alkaline. The hydrophilicity of the proteins encoded by the *MeMED* genes ranged from −0.948 to 0.227.

**Figure 1:**
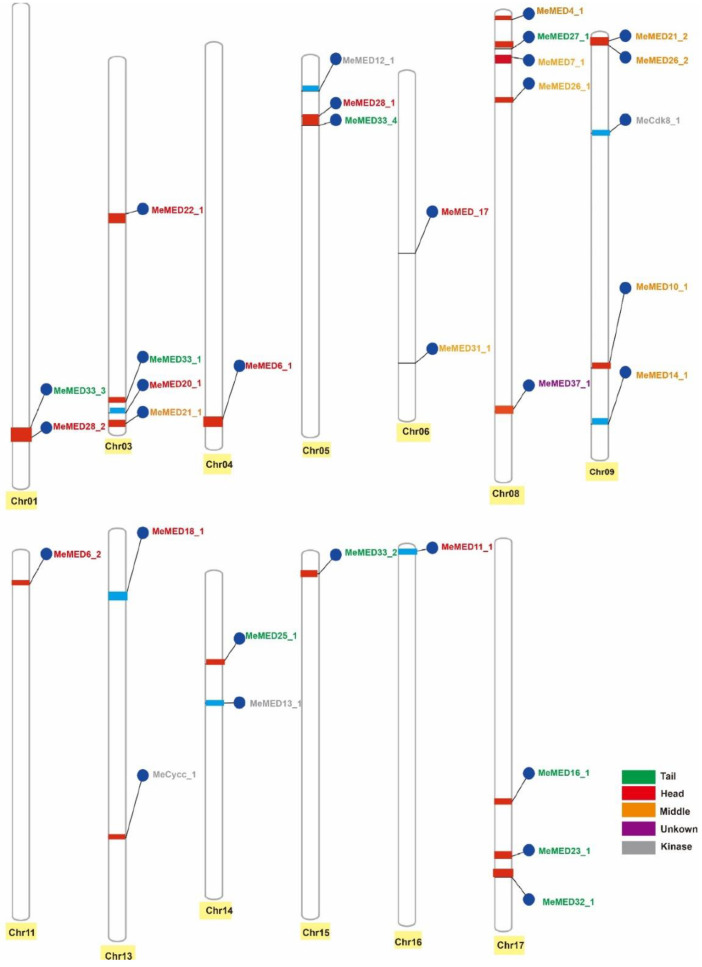
Chromosomal localization and distribution of *MeMED* Gene in Cassava. The yellow color was used solely to enhance the visibility of the chromosomes, providing a clear contrast. It does not convey any additional specific meaning or indication.

The Mediator subunits in cassava were categorized according to the Arabidopsis Mediator modules. Among them, nine subunits were assigned to the head module, nine to the middle module, nine to the tail module, four to the kinase module, and one subunit could not be classified (Table 1). Chromosomes 8 and 9 each contained a relatively large number of genes, with five genes on each chromosome. Chromosomes 4, 11, 15, and 16 each harbored one *MeMED* gene. Additionally, chromosomes 1, 5, and 9 each contained a gene cluster, with the following gene pairs: *MeMED33_3* and *MeMED28_2*, *MeMED28_1* and *MeMED33_4*, and *MeMED21_2* and *MeMED26_2* (Figure 1).

### 2.2. Phylogenetic Analysis of Cassava MeMED Proteins

**Figure 2:**
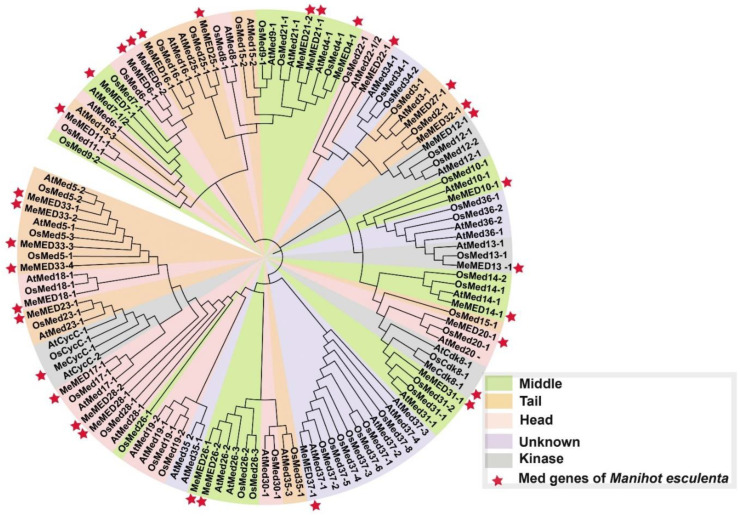
Phylogenetic analysis of MeMED proteins from three plant species, namely *Manihot esculenta*, *Arabidopsis thaliana*, and *Oryza sativa*. The phylogenetic tree was constructed by using mega 7 with the maximum likelihood method and a bootstrap value of 1000 replicates. The different colors in the tree represent different MED modules. The red-colored star represents the MED genes in *Manihot esculenta.*

We constructed a phylogenetic tree for 125 MeMED proteins (42 from *Arabidopsis*, 32 from *Manihot esculenta* Crantz, and 51 from *Oryza sativa*) to study the evolutionary relationships of MeMED proteins across these species (Figure 2). Based on their evolutionary divergence, the MeMED proteins in cassava can be classified into three subclades. The first subclade includes nine proteins, namely MeMED11_1, MeMED7_1, MeMED6_1, MeMED6_2, MeMED16_1, MeMED25_1, MeMED21_2, MeMED21_1, and MeMED4_1. Among them, three pairs of orthologous MED members are shared between cassava and rice as follows: MeMED11_1 and OsMED11_1, MeMED25_1 and OsMED25_1, and MeMED4_1 and OsMED4_1. The second subclade includes 10 proteins, namely MeMED22_1, MeMED27_1, MeMED32_1, MeMED12_2, MeMED10_1, MeMED13_1, MeMED14_2, MeMED20_1, MeMED31_1, and MeCdk8_1. Among these, one pair of orthologous MED members is shared between cassava and rice, which is MeMED13_1 and OsMED13_1. The third subclade includes 13 proteins, with two pairs of orthologous MED members shared between cassava and rice as follows: MeMED23_1 and OsMED23_1, and MeMED17_1 and OsMED17_1. Based on the Ka/Ks ratio, the selective pressure on genes can generally be inferred: a Ka/Ks ratio greater than one suggests positive selection, a ratio less than one indicates purifying selection, and a ratio of one is associated with neutral selection. The Ka/Ks ratios for the *MeMED* gene pairs range from 0.08 to 0.59, reflecting that all the *MeMED* gene pairs are under negative or purifying selection (Ka/Ks < 1) (Table S1). These results indicate that purifying selection has played a role in preserving the conservation of the *MeMED* gene structure during domestication or evolutionary processes.

**Figure 3:**
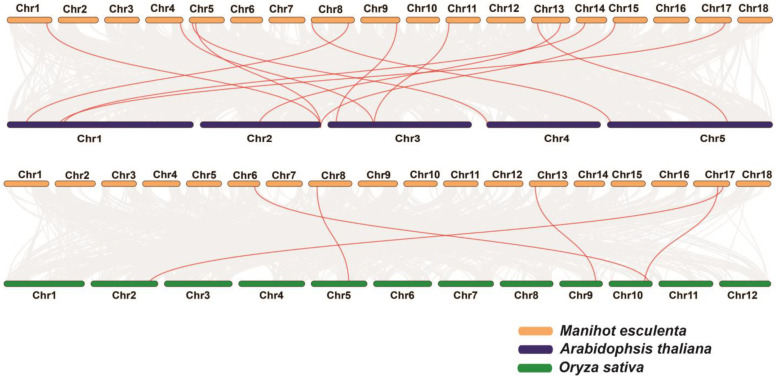
Collinear analysis between the cassava *MeMED* genes and the genomes of *Arabidopsis* and *Oryza sativa*.

Gene synteny analysis across species revealed that 12 *MeMED* members in cassava exhibit synteny with *AtMED* members in *Arabidopsis*. These cassava genes are distributed on chromosomes 1, 4, 5, 8, 9, 11, 13, 14, 15, and 17, while the corresponding Arabidopsis genes are located on five chromosomes (Figure 3). Additionally, five *MeMED* members in cassava show synteny with *OsMED* members in *Oryza sativa*. These cassava genes are located on chromosomes 6, 8, 13, and 17, while the corresponding rice genes are distributed on chromosomes 2, 5, 9, and 10 (Figure 3).

### 2.3. Analysis of Gene Structure and Cis-Acting Elements of the Cassava MeMED Genes

**Figure 4:**
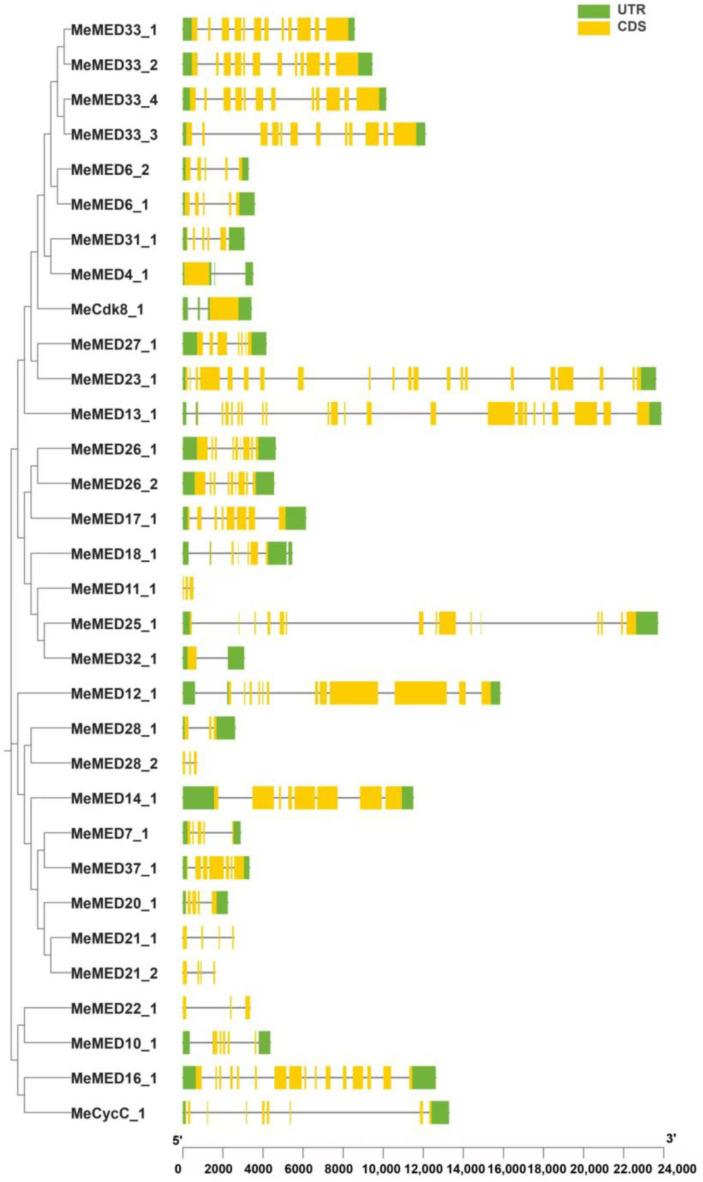
Gene structure analysis of the cassava *MeMED* gene family.

Based on gene sequence similarity, we classified the 32 *MeMED* genes into three distinct groups. The largest group contains 19 *MeMED* genes, while the other two groups consist of nine and four *MeMED* genes, respectively (Figure 4). The number of exons in *MeMED* genes ranges from 1 to 23. For example, *MeMED4_1*, *MeCdk8_1*, and *MeMED32_1* each contain a single exon, while *MeMED13_1* contains 23 exons (Figure 4). These results indicate significant functional divergence among the *MeMED* genes during their evolutionary process.

**Figure 5:**
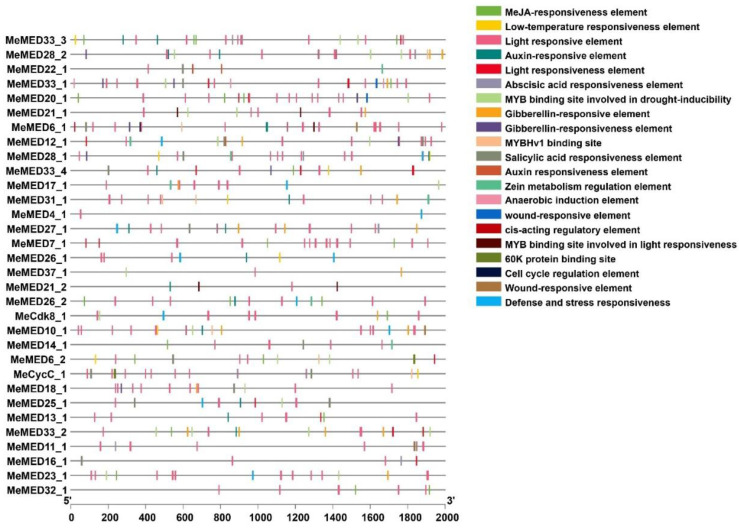
Evaluation of cis-regulatory elements in the *MeMED* gene promoters’ regions. The colored boxes denote different cis-regulatory elements, each linked to specific biological responses or functions.

Cis-regulatory elements play a crucial role in regulating gene expression. To identify the types of regulatory elements, we analyzed the 2 kb promoter sequences of all *MeMED* genes using the PlantCARE database. The promoter regions of *MeMED* genes primarily contain four categories of *cis*-regulatory elements, including light-responsive, hormone-responsive, stress-inducible, and core elements/binding sites (Figure 5). Most *MeMED* genes are associated with light-responsive elements, such as G-Box, GA-motif, GT1-motif, GATA-motif, TCT-motif, ACE, I-box, Gap-box, TCCC-motif, AE-box, 3-AF1 binding site, chs-CMA1a, Sp1, LAMP-element, and ATCT-motif. Hormone-responsive elements include abscisic acid-responsive elements (ABRE), methyl jasmonate-responsive elements (TGACG-motif, CGTCA-motif), salicylic acid-responsive elements (TCA-element), auxin-responsive elements (AuxRE, TGA-element), and gibberellin-responsive elements (GARE-motif, P-box, TATC-box). Stress-inducible elements include low-temperature-responsive elements (LTR) and defense/stress-related elements (TC-rich repeats, WUN-motif). Core elements and binding sites include O_2_-site, CCAAT-box, and MBS (Table S2). These findings suggest that *MeMED* genes may play significant roles in plant growth, development, and stress resistance.

### 2.4. Expression Profiles of MeMED Genes in Different Tissues of Cassava Based on RNA-Seq

**Figure 6:**
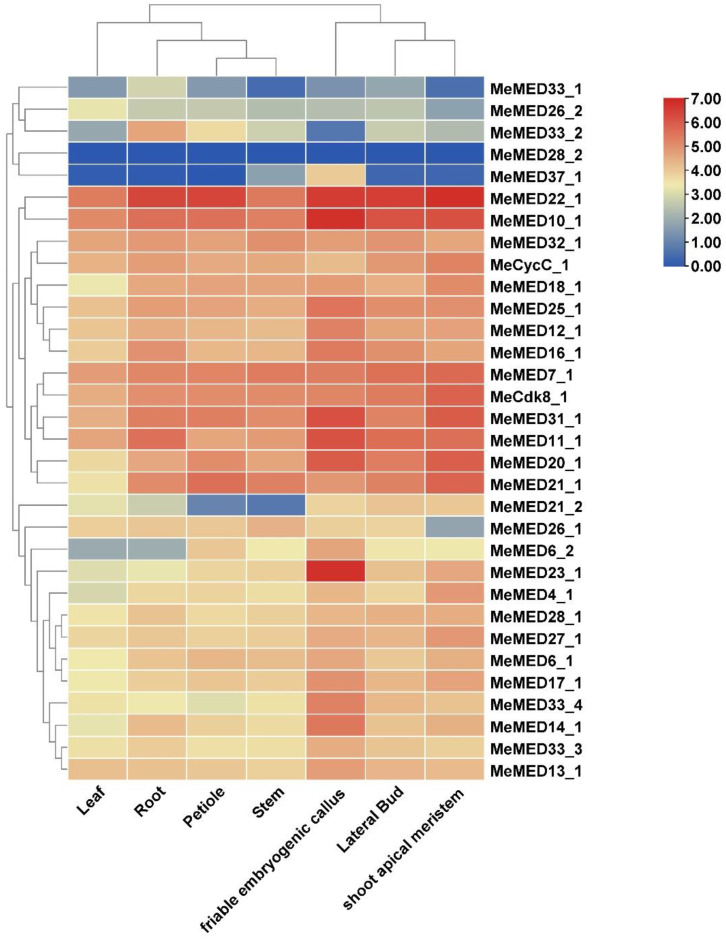
Tissue-specific expression analysis of the *MeMED* gene family in cassava. The red color represents higher expression, the yellow color indicated low expression, whereases the blue color indicated no expression in the RNA-seq expression data.

To explore the potential function of the *MeMED* gene in cassava, we analyzed RNA-seq data from the GEO database to examine the expression patterns of *MeMED* gene family members across seven different tissues. Transcriptome analysis revealed that 32 *MeMED* genes exhibited detectable transcriptional abundance across seven different organs, with significant expression differences observed among genes in various tissues (Figure 6). Specifically, *MeMED22_1*, *MeMED10_1*, *MeMED7_1*, and *MeMED11_1* were highly expressed in leaves; *MeMED22_1*, *MeMED10_1*, and *MeMED11_1* showed high expression levels in roots; *MeMED22_1*, *MeMED10_1*, *MeMED32_1*, *MeCycC_1*, *MeMED7_1*, and *MeMED11_1* were highly expressed in petioles; *MeMED22_1*, *MeMED10_1*, and *MeMED21_1* were highly expressed in stems; and *MeMED23_1*, *MeMED22_1*, and *MeMED10_1* were highly expressed in friable embryogenic callus. Notably, *MeMED22_1* and *MeMED10_1* were highly expressed in shoot apical meristems. Additionally, *MeMED28_2* and *MeMED37_1*, except in friable embryogenic callus, showed relatively low expression across the seven organs, indicating that they may be non-functional (Figure 6). These findings suggest that members of the *MeMED* gene family may exhibit functional differentiation in cassava.

### 2.5. Expression Analysis of MeMED Genes in Response to Abiotic Stresses

**Figure 7:**
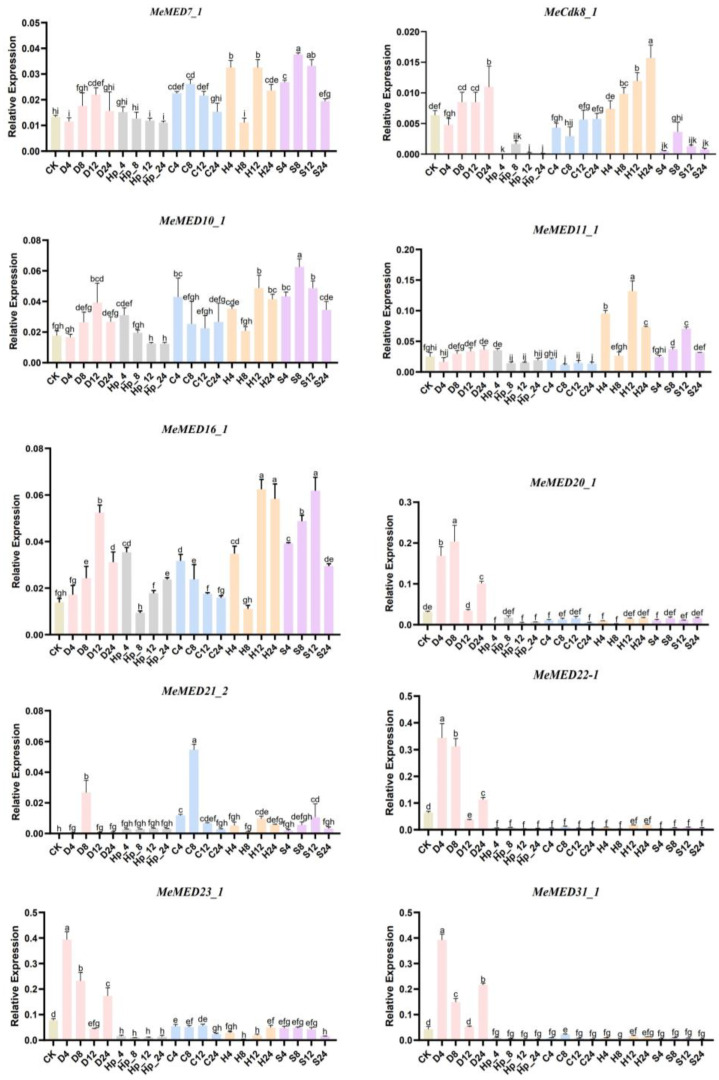
Expression patterns of selected *MeMED* genes under different abiotic stresses. Data are presented as means ± standard errors. Statistically significant differences are indicated by asterisks (*p* ≤ 0.05). CK, control; D, drought; HP, exogenous hydrogen peroxide; C, cold; H, heat; S, salt. Time intervals of 4, 8, 12, and 24 h are denoted by the numbers 4, 8, 12, and 24, respectively. Samples collected at 0 h served as the control (CK). Statistical significance was determined using a one-way ANOVA followed by Tukey’s HSD post hoc test. Different letters indicate significant differences (*p* < 0.05) within each group.

Based on the RNA-seq expression profile, we selected 10 *MeMED* genes that are highly expressed across various tissues to analyze their expression in response to abiotic stresses. To investigate the expression of these genes under abiotic stresses (drought, exogenous hydrogen peroxide, cold, heat, and salt), tissues were analyzed at 4 h, 8 h, 12 h, and 24 h after treatment using RT-qPCR. The results indicated that the 10 *MeMED* genes were relatively sensitive to drought, exogenous hydrogen peroxide, cold, heat, and salt treatments (Figure 7). For example, the expression of *MeCdk8_1* significantly increased from 4 h to 24 h hours under drought and heat treatments, whereas it significantly decreased under exogenous hydrogen peroxide treatment. The expression of *MeMED7_1* was significantly upregulated after 4 h of salt treatment, reaching its highest level at 8 h. The expression of *MeMED16_1* was significantly upregulated at 4 h post-cold treatment and then decreased to its lowest level at 24 h. *MeMED11_1* showed a significant upregulation at 12 h after heat treatment. Interestingly, *MeMED20_1*, *MeMED22_1*, *MeMED23_1*, and *MeMED31_1* exhibited similar expression patterns after drought treatment, with significant upregulation, while their expression levels decreased under exogenous hydrogen peroxide, cold, heat, and salt treatments. Furthermore, the expression of *MeMED21_1* displayed a unique pattern, significantly increasing after 8 h of drought and cold treatments. These findings suggest that the *MeMED* genes may exhibit functional differentiation in response to various stress conditions.

## 3. Discussion

Given the crucial role of *MeMED* genes in various physiological processes, including responses to biotic and abiotic stresses [16], it is important to investigate their potential functions in cassava. Currently, *MeMED* genes have been identified and phylogenetically analyzed in only a few plants [7,19]. However, *MeMED* genes have not yet been identified in cassava. In this study, we identified 32 *MeMED* genes in the cassava genome (Table 1 and Figure 1). This number is lower than that found in *Arabidopsis*, rice, and asparagus bean. The variation in the number of *MeMED* genes across species not only reflects differences in their evolutionary history but may also be linked to factors such as genome characteristics, ecological adaptation, and stress response mechanisms.

### 3.1. Phylogenetic Relationships and Evolution of the MeMED Gene Family

Analyzing the phylogenetic relationships and evolutionary patterns of gene families across different species provides valuable insights into gene conservation, functional diversification, and the evolutionary history of these genes. In our phylogenetic analysis of the 32 *MeMED* genes in cassava, we compared them with homologous genes from *Arabidopsis* and rice. The results revealed that the cassava *MeMED* gene family is closely related to the *MED* gene families of other plant species, suggesting a conserved evolutionary history. For example, three pairs of orthologous *MED* genes were identified between cassava and rice, namely *MeMED11_1* and *OsMED11_1*, *MeMED25_1* and *OsMED25_1*, and *MeMED4_1* and *OsMED4_1* (Figure 2).

Furthermore, the presence of orthologous gene pairs between cassava and rice strengthens the hypothesis of a shared ancestral origin for these genes. Despite the divergence of cassava (Euphorbiaceae) and rice (Poaceae) into separate plant families, the observed synteny between the two species suggests that the *MeMED* gene family in both plants likely originated from a common ancestor. This highlights the conserved nature of the *MeMED* family and provides insights into its evolutionary conservation across distantly related species.

### 3.2. Regulation of MeMED Genes by Cis-Acting Elements

Analyzing the cis-regulatory elements in the promoter regions of plant genes is essential for understanding the mechanisms that regulate gene expression [24]. The analysis of cis-regulatory elements in the promoter regions of *MeMED* genes revealed that many of these genes are associated with light-responsive, hormone-responsive, and stress-inducible elements (Figure 5). These findings suggest that *MeMED* genes play crucial roles in regulating plant growth, development, and responses to various environmental stresses. The observation that most *MeMED* genes contain light-responsive elements further supports the possibility that they are involved in regulating processes that respond to changing environmental light conditions, such as photomorphogenesis or circadian rhythm regulation.

The presence of stress-related elements, such as low-temperature-responsive (LTR) motifs and defense-/stress-related sequences, underscores the potential importance of these genes in cassava’s adaptation to abiotic stresses, particularly low temperature. This is especially relevant considering the increasing frequency and intensity of climate-induced stresses affecting crop production globally. In other plants, some *MeMED* genes associated with low temperature have been well studied. For example, *AtMED16* in *Arabidopsis* is involved in the CBF (C-repeat binding factor) pathway, which is a central mechanism in cold stress response [25]. Furthermore, *AtMED16*, along with *AtMED14* and *AtMED2*, is required for the expression of certain cold-responsive genes that are induced by low temperatures. However, it is important to note that not all cold-responsive genes are regulated by these factors, indicating a complex and specific regulation of gene expression under cold stress [16]. This emphasizes the multifaceted role of *MeMED* genes, which may function similarly in cassava, regulating a subset of cold-responsive genes while leaving others unaffected.

### 3.3. Expression Characteristics of MeMED Genes in Response to Stress

The Mediator complex plays a pivotal role as a co-activator in transcriptional regulation in plants, participating in nearly all gene transcription processes. Numerous studies have emphasized its involvement in abiotic stress responses, highlighting the complex’s role in modulating stress-related genes [7,26]. In this study, we investigated the expression changes in ten cassava *MeMED* genes under various stress treatments (Figure 7). Most of these genes exhibited significant alterations in expression throughout the stress treatments, underscoring the crucial role of *MeMED* genes in cassava’s response to environmental stresses. Previous studies have confirmed the crucial role of CDK8 in responses to drought, salt, and cold stresses [14,17,20]. Our study yielded similar results, demonstrating that *MeCdk8_1* exhibited a significant increase in expression under both drought and heat stress, suggesting its potential involvement in regulating the cell cycle and stress responses. Notably, its expression was markedly lower under hydrogen peroxide treatment, indicating a possible role in oxidative stress regulation. These findings highlight the versatility of *MeCdk8_1* in responding to various stress signals and suggest that it may play a similarly critical role in multiple stress responses in cassava. Further research is needed to elucidate its precise function and underlying mechanisms in stress tolerance.

Interestingly, *MeMED16_1* was significantly upregulated at 4 h post-cold treatment before its expression declined to its lowest level at 24 h, suggesting its involvement in early cold stress signaling. These findings align with previous studies, which demonstrated that *At-MED16* detects nuclear signals, participates in cold response pathways, and enhances nuclear replication and cell growth in *Arabidopsis* [25,27]. Moreover, our results are consistent with those of *VuMED16* in asparagus bean, which has been shown to modulate cell wall responses to cold stress [19].

Multiple studies in Arabidopsis have also reported the role of *AtMED* genes in salt stress, including *AtMED25* [12], *AtMED18* [13], and *Atcdk8* genes [14]. In *Arabidopsis*, *AtMED16*, *AtMED14*, and *AtMED2*, as essential Mediator subunits, participate in the activation of ABRE motif-mediated regulation. Mutant plants lacking these genes are insensitive to ABA-induced growth inhibition but exhibit a significantly higher water loss rate in detached leaves compared to the wild-type, underscoring the specific role of these genes in drought response [16]. In contrast, *MeMED11_1* showed a marked upregulation after heat stress, further suggesting that it may be implicated in the heat stress response. The consistent upregulation of *MeMED20_1*, *MeMED22_1*, *MeMED23_1*, and *MeMED31_1* under drought conditions, followed by a decline in expression under other stresses, indicates that these genes may play more specific roles in drought tolerance and possibly share common regulatory pathways related to water deficit.

Of particular interest is *MeMED21_1*, which exhibited a unique expression pattern: it significantly increased after 8 h of drought and cold treatments, suggesting that it may play a dual role in mediating responses to both drought and cold stresses. This dual regulation could enhance cassava’s adaptability to multiple environmental stresses, potentially contributing to its stress tolerance. These findings are consistent with previous research on *Arabidopsis*, where *AtMED25* has been shown to regulate responses to both drought and salt stresses [12]. Further research on *MeMED21_1* could elucidate its precise molecular mechanisms and provide valuable insights for improving stress resilience in cassava cultivation.

## 4. Material and Methods

### 4.1. Mining of MED Family in Manihot esculenta

In our study, we searched the TAIR database to obtain the MED protein sequences for *Arabidopsis* (https://www.arabidopsis.org/, accessed on 2 July 2024) [28]. These sequences were used as query sequences and were blasted against the cassava genome (*Manihot esculenta* v8.1) available online at phytozome (https://phytozome-next.jgi.doe.gov/info/Mesculenta_v8_1, accessed on 1 July 2024) using the BlastP tool (https://phytozome-next.jgi.doe.gov/blast-search, accessed on 2 July 2024). After removing duplicates, the non-redundant protein sequences were analyzed using SMART (http://smart.embl-heidelberg.de/, accessed on 2 July 2024) and NCBI conserved domain searches (https://www.ncbi.nlm.nih.gov/Structure/cdd/wrpsb.cgi, accessed on 4 July 2024) [29]. The final MED proteins were renamed according to their physical positions in the cassava genome (*Manihot esculenta* v8.1). The MED modules (head, tail, middle, unknown, and kinase) were named according to the *A. thaliana* MED genes module [7]. Additionally, the physicochemical properties of the 32 MeMED proteins, including protein length (aa), theoretical isoelectric point (pI), molecular weight (kDa), and grand average of hydropathicity (GRAVY), were computed using ProtParam (https://web.expasy.org/protparam/, accessed on 8 July 2024) [30].

### 4.2. Phylogeny of Cassava MED Proteins

To construct the phylogenetic tree of MeMED proteins, the protein sequences of cassava, *A. thaliana*, and *Oryza sativa* were first aligned using MEGA software (Version 7) [31]. The maximum likelihood tree was then generated using IQ-TREE software, version 2.0 (http://www.iqtree.org, accessed on 11 July 2024) [32] with bootstrap values set to 1000 replicates. The resulting phylogeny was further refined using ITOL (https://itol.embl.de/, accessed on 14 July 2024) [33].

### 4.3. Gene Structural Analysis of Cassava MED Proteins

The intron and exon organization of the *MeMED* genes was predicted using TBtools (version V2.056) [34]. To identify the cis-acting elements within the promoters of the MeMED genes, a 2 kb sequence upstream of the start codon was submitted to the PlantCARE website [35]. The graphical representation of the cis-regulatory elements in *MeMED* genes was generated using TBtools.

### 4.4. Evolutionary Analysis of MeMED Genes

The synteny relationships among *Cassava*, *Oryza sativa*, and *Arabidopsis* were assessed using MCScanX, MCScanX software, version 1.0 and visualization was performed with TBtools (v2.012) [36]. Additionally, the nonsynonymous (Ka) and synonymous (Ks) substitution rates between gene pairs were calculated. The divergence time (T, in millions of years ago; MYA) was estimated using the following formula: T = Ks/2R, where R = 15 × 10^−8^ represents the rate of synonymous substitutions per site per year [37].

### 4.5. Expression Analyses of MeMED Genes in Different Tissues

The Bart Lab Cassava Atlas database (https://shiny.danforthcenter.org/cassava_atlas/, accessed on 28 July 2024) was searched to obtain the expression data for the *MeMED* genes. Temporal and spatial expression patterns of the MeMED proteins were evaluated using RNA-seq data from various cassava tissues, including the root, stem, leaf, and petiole, at different developmental stages. The heatmap was generated using TBtools. The FPKM values were calculated as described in previous studies [38].

### 4.6. RNA Extraction, cDNA Synthesis, and RT-qPCR Analysis

Total RNA was extracted using the RNAprep Pure Plant Kit (Lot no. Q5510, Tiangen, Beijing, China), following the manufacturer’s instructions. The first complementary DNA strand (cDNA) was synthesized using the HiScript 1st Strand cDNA Synthesis Kit V221 (Vazyme Biotech Co., Ltd., Sanya, China). RT-qPCR was performed using ChamQ™ SYBR RT-qPCR Master Mix (Vazyme Biotech Co., Ltd., Sanya, China). Each reaction had a total volume of 20 μL, comprising 0.4 μL forward primer, 0.4 μL reverse primer, 2 μL cDNA, 10 μL SYBR mix, and 7.2 μL ddH_2_O. The cycling program included an initial denaturation step at 95 °C for 30 s, followed by 40 cycles of denaturation at 95 °C for 10 s and annealing/extension at 60 °C for 30 s. *Me*-actin was used as an internal control. The data were analyzed using the 2^−ΔΔCt^ method [39]. The primers for the selected *MeMED* genes are listed in Table S3. The heatmap was generated using GraphPad Prism 8 software.

### 4.7. Plant Material and Stress Treatment Conditions

In this study, the typical cultivated cassava variety SC9 (South China 9) was used for stress treatments. The SC9 seeds were obtained from the National Cassava Germplasm Repository (NCGR) at the Tropical Crops Genetic Resources Institute, Chinese Academy of Tropical Agricultural Sciences, HaikouHainan Province, China. Vigorous seeds were selected and sterilized with a 10% hypochlorous acid solution for 5 min. The seeds were then grown on a water-saturated filter paper in a growth chamber (25 °C day/night with a 16 h/8 h light/dark cycle), after which the plants were transferred to pots containing soil. For stress treatments, the plants were subjected to 300 mM NaCl for salt stress, 20% PEG6000 for drought stress, 4 °C for cold stress, 42 °C for heat stress, and 10% hydrogen peroxide (H_2_O_2_) for oxidative stress. Samples were collected at 0 (control), 4, 8, 12, and 24 h post-treatment. Three biological replicates were performed for each treatment. All samples were rapidly frozen in liquid nitrogen and stored at −80 °C for subsequent expression analysis.

## 5. Conclusions

This study provides the first genome-wide analysis of the *MeMED* gene family in cassava. We identified 32 *MeMED* genes and examined their basic features, gene structure, evolutionary relationships, and expression patterns. RT-qPCR analysis showed significant changes in the expression of the selected 10 *MED* genes, including *MeMED7-1*, *MeCdk8-1*, *MeMED10-1*, *MeMED11-1*, *MeMED16-1*, *MeMED20-1*, *MeMED21-1*, *MeMED22-1*, *MeMED23-1*, *and MeMED31-1* (under various abiotic stresses, suggesting their potential role in enhancing cassava’s stress tolerance). The *MeMED21_1*, which exhibited a unique expression pattern towards abiotic stress, could offer new strategies to improve cassava resilience. These findings have a significant implication for climate change and sustainable agriculture. The identified genes in our study may contribute to new cassava varieties that may show more tolerance towards the harsh environmental conditions, which are becoming more prevalent due to climate change. This study can help to ensure food production and contribute to the sustainable production of cassava. Overall, our research provides valuable insights into the *MeMED* gene family and lays the groundwork for future studies on important crops like cassava.

## Figures and Tables

**Table 1:** Information on the *MeMED* genes in cassava.

	Gene Name	Chr	CDS (bp)	Protein Length (A.A)	Protein Molecular Weight (KDa)	pI	Gravy	Mediator Module
Manes.01G210100.1	*MeMED33_3*	1	3987	1329	144.55	6.71	0.169	Tail
Manes.01G220201.1	*MeMED28_2*	1	318	106	12.41	6.13	−0.185	Head
Manes.03G077300.1	*MeMED22_1*	3	477	159	17.00	6.12	−0.226	Head
Manes.03G185600.9	*MeMED33_1*	3	3969	1323	144.45	6.68	0.227	Tail
Manes.03G199800.1	*MeMED20_1*	3	666	222	25.36	6.08	−0.266	Head
Manes.03G210700.11	*MeMED21_1*	3	405	135	14.69	4.4	−0.37	Middle
Manes.04G136400.1	*MeMED6_1*	4	741	247	27.51	5.18	−0.378	Head
Manes.05G034300.10	*MeMED12_1*	5	6798	2266	250.60	8.92	−0.27	Kinase
Manes.05G061600.1	*MeMED28_1*	5	438	146	17.12	5.78	−0.886	Head
Manes.05G073300.1	*MeMED33_4*	5	3993	1331	143.11	7.22	0.185	Tail
Manes.06G049700.1	*MeMED17_1*	6	1998	666	74.30	5.65	−0.3	Head
Manes.06G129600.1	*MeMED31_1*	6	621	207	23.70	9.28	−0.637	Middle
Manes.08G002200.1	*MeMED4_1*	8	1239	413	44.99	4.97	−0.542	Middle
Manes.08G033200.3	*MeMED27_1*	8	1263	421	46.41	8.76	−0.292	Tail
Manes.08G039900.1	*MeMED7_1*	8	507	169	19.64	9.16	−0.675	Middle
Manes.08G061400.1	*MeMED26_1*	8	1431	477	53.66	5.56	−0.948	Middle
Manes.08G118700.1	*MeMED37_1*	8	1983	661	73.14	5.28	−0.463	Unknow
Manes.09G000400.6	*MeMED21_2*	9	405	135	14.74	4.42	−0.407	Middle
Manes.09G002800.2	*MeMED26_2*	9	1443	481	54.11	5.94	−0.844	Middle
Manes.09G101000.3	*MeMED10_1*	9	564	188	20.41	5.23	−0.384	Middle
Manes.09G152800.6	*MeMED14_1*	9	5478	1826	197.87	7.96	−0.202	Middle
Manes.11G030300.3	*MeMED6_2*	11	744	248	27.74	5.2	−0.468	Head
Manes.13G095300.2	*MeMED18_1*	13	690	230	24.59	6.71	0.137	Head
Manes.14G098600.1	*MeMED25_1*	14	2511	837	89.26	8.79	−0.343	Tail
Manes.14G121800.7	*MeMED13_1*	14	5925	1975	213.55	5.64	−0.208	Kinase
Manes.15G022100.1	*MeMED33_2*	15	3972	1324	143.98	6.29	0.185	Tail
Manes.16G001300.1	*MeMED11_1*	16	351	117	13.26	5.78	−0.521	Head
Manes.17G043400.1	*MeMED16_1*	17	3756	1252	135.29	6.1	−0.232	Tail
Manes.17G082000.1	*MeMED23_1*	17	4839	1613	180.90	6.64	−0.08	Tail
Manes.17G099700.2	*MeMED32_1*	17	441	147	15.40	4.69	−0.098	Tail
Manes.13G050900.1	*MeCycC_1*	13	762	254	29.76	6.54	−0.098	Kinase
Manes.09G052700.5	*MeCdk8_1*	9	1434	478	53.00	9.24	−0.491	Kinase

## Data Availability

The authors confirm that the data supporting the findings of this study are available within the article and its Supplementary Materials.

## Supplementary Materials

The following supporting information can be downloaded at: https://www.mdpi.com/article/10.3390/ijms26041666/s1.
